# Tropocollagen springs allow collagen fibrils to stretch elastically

**DOI:** 10.1016/j.actbio.2022.01.041

**Published:** 2022-04-01

**Authors:** James S. Bell, Sally Hayes, Charles Whitford, Juan Sanchez-Weatherby, Olga Shebanova, Nick J. Terrill, Thomas L.M. Sørensen, Ahmed Elsheikh, Keith M. Meek

**Affiliations:** a School of Optometry and Vision Sciences, Cardiff University, Maindy Road, Cathays, Cardiff CF24 4HQ, United Kingdom; b Cardiff Institute for Tissue Engineering and Repair (CITER); c The Manufacturing Technology Centre Ltd., Knowledge Quarter, Liverpool, United Kingdom; d Diamond Light Source Ltd, Diamond House, Harwell Science & Innovation Campus, Didcot, Oxfordshire OX11 0DE, United Kingdom; e Department of Biological and Chemical Engineering, Aarhus University, Gustav Wieds Vej 10, 8000 Aarhus C, Denmark; f School of Engineering, University of Liverpool, The Quadrangle, Brownlow Hill, Liverpool L69 3GH, United Kingdom; g NIHR Biomedical Research Centre for Ophthalmology, Moorfields Eye Hospital NHS Foundation Trust and UCL Institute of Ophthalmology, United Kingdom

**Keywords:** Collagen, Cornea, X-ray scattering, Biomechanics, Crimp

## Abstract

The mechanical properties of connective tissues are tailored to their specific function, and changes can lead to dysfunction and pathology. In most mammalian tissues the mechanical environment is governed by the micro- and nano-scale structure of collagen and its interaction with other tissue components, however these hierarchical properties remain poorly understood. In this study we use the human cornea as a model system to characterise and quantify the dominant deformation mechanisms of connective tissue in response to cyclic loads of physiological magnitude. Synchronised biomechanical testing, x-ray scattering and 3D digital image correlation revealed the presence of two dominant mechanisms: collagen fibril elongation due to a largely elastic, spring-like straightening of tropocollagen supramolecular twist, and a more viscous straightening of fibril crimp that gradually increased over successive loading cycles. The distinct mechanical properties of the two mechanisms suggest they have separate roles in vivo. The elastic, spring-like mechanism is fast-acting and likely responds to stresses associated with the cardiac cycle, while the more viscous crimp mechanism will respond to slower processes, such as postural stresses. It is anticipated that these findings will have broad applicability to understanding the normal and pathological functioning of other connective tissues such as skin and blood vessels that exhibit both helical structures and crimp.

**Statement of significance:**

The tropocollagen spring mechanism allows collagen fibrils from some tissues to elongate significantly under small loads, and its recent discovery has the potential to change our fundamental understanding of how tissue deforms. This time-resolved study quantifies the contribution of the spring mechanism to the local strain in stretched tissue and compares it to the contribution associated with the straightening of fibril waviness, the widely accepted primary low-load strain mechanism. The spring mechanism contributed more to the local tissue strain than fibril straightening, and was found to be elastic while fibril straightening was more viscous. The results suggest that the viscoelastic behaviour of a biomaterial is controlled, at least in part, by the relative amount of fibril-scale crimp and tropocollagen supramolecular twist.

## Graphical abstract

**Figure:**
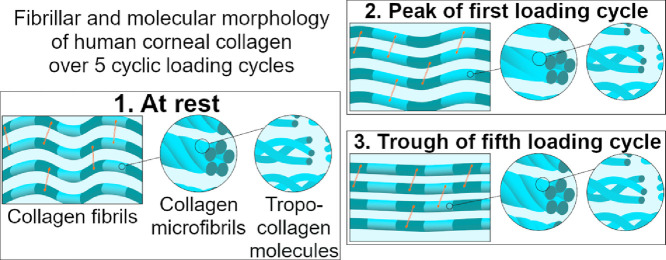

## 1 Introduction

Collagen is the most abundant protein in the mammalian body and has the primary role of providing mechanical support to the extracellular matrix. This role is fulfilled by forming fibrils or lattices that are mechanically stiff and tough, and which interact with other interstitial proteins and fluid to imbue the local tissue with requisite mechanical environments [1,2]. The mechanical properties of collagen are hierarchical; [3] the type I tropocollagen molecule has an elastic modulus ranging between 2.9 GPa and 9 GPa in tension [4], while larger structures such as fibrils and tendon fascicles are over an order of magnitude more compliant [5]. The drop in stiffness with increased hierarchical scale is associated with an increased number of deformation mechanisms [6].

Corneal collagen is exceptional; the fibrils are of uniform diameter, they are thin compared to those in other tissues, and they exist in a crystalline arrangement that confers transparency, a quality that is essential to the function of the cornea as the primary lens of the eye [7]. In addition to being transparent, the cornea must also maintain a precise curvature for optimal vision and in doing so, minimise distortions associated with intraocular pressure fluctuations, blinking and eye movement, which can cause intraocular pressure to increase by over 100% [8]. These factors must be offset, however, with a certain amount of compliance across the entire corneoscleral shell to accommodate changes in intraocular fluid volume [9], thus avoiding pressure spikes and associated retinal damage common in glaucoma [10]. Given that the stiffness of corneal collagen fibrils is generally regarded as being too high for physiological loads to cause any appreciable strain, the ability of the cornea (and most other collagenous tissues) to deform in vivo is usually attributed to fibril uncrimping (straightening of fibril waviness). However, assuming a fibril is composed of an effectively inextensible material, mechanical models of fibril crimp [11] cannot account for the strains measured in inflation experiments (strains of ∼2.5%) [12] when using measured corneal crimp values from the literature showing tortuosities – ratios of straightened to crimped fibril length – of 0.5–1%. [13] There is therefore a compelling case for a smaller-scale mechanism that regulates the mechanical response of the cornea to load.

**Fig. 1:**
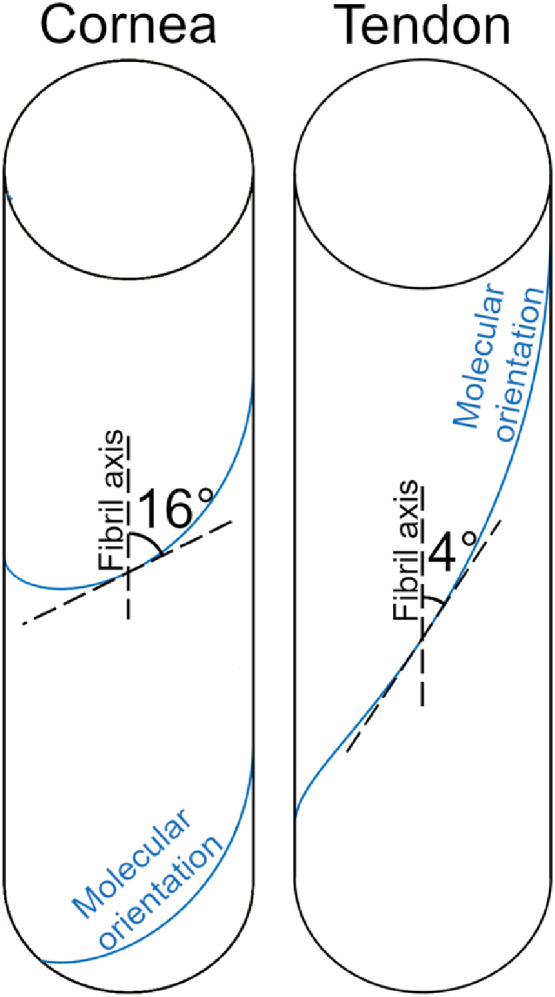
The supramolecular twist angle in a tissue such as cornea, skin, nerve, tendon sheath or the arterial wall (left) versus that of a tissue such as tendon or ligament (right).

X-ray scattering has been used extensively to study the nanostructure of the cornea in health [14,15] and disease [16], to inform mechanical models [17] and evaluate treatments [18]. The crystalline arrangement of collagen fibrils in the cornea makes it an ideal test bed for the study of collagen mechanics in general. An initial exploration using x-ray scattering techniques showed that, under loads of physiological magnitude, the primary deformation mechanism in corneal tissue is a spring-like straightening of tropocollagen supramolecular twist [19]. This twist occurs at a scale larger than the tropocollagen triple helix, but smaller than fibrillar crimp. This confirmed predictions made via atomistic mechanical modelling [20] of collagen type I microfibrillar architecture determined using crystallographic techniques [21]. This spring-like deformation mechanism is likely to be a major contributor to the physiological functioning of connective tissues containing fibrillar collagen with naturally high supramolecular twist angle (15°−17° [19,22,23], see Fig. 1) such as cornea, skin, arteries, and the sheaths of nerves and tendons. These tissues experience relatively small, planar loads and contain fine fibrils with d-periods of approximately 65 nm (the d-period is a longitudinal feature of the collagen fibril associated with the precise axial stagger in the arrangement of tropocollagen molecules, which gives rise to a banding pattern in electron microscopy and meridional diffraction peaks in x-ray scattering). Tissues with low supramolecular twist angles (approximately 4°), such as tendon and ligament, contain parallel fibrils of variable diameter with d-periods of approximately 67 nm [24,25], which resist larger, uniaxial loads [26].

It has been suggested that to assemble fibrils with a thin and regular diameter as in the cornea, a high supramolecular twist angle may be required to ensure the thermodynamic stability of the tissue [27,28]. In addition to this, it is thought that another likely physiological role of the tropocollagen supramolecular twist (herein referred to as the “tropocollagen spring”) may be to add elastic compliance to tissues that do not typically experience high forces [19,24]. Elastic deformations are recoverable with minimal energy loss, due to efficient mechanical energy storage [29]. In contrast, under high loads, collagen exhibits a well-defined viscous response which dampens deformations and minimises shock [30]. The inherent viscoelasticity of soft tissue [31] complicates the study of its biomechanical properties, as the way in which a specimen deforms is dependant upon its loading history. This difficulty is reduced somewhat by artificially preconditioning a specimen prior to biomechanical testing, in order to remove most of the non-recoverable strains and bring the tissue to a defined reference point [32]. A limitation of this approach is that it perturbs the tissue from its natural state. Furthermore, each hierarchical mechanism exhibits varying time- and strain-dependant viscoelastic properties [33,34], meaning a specimen can never be “perfectly preconditioned”.

It is still not known how the structure of collagen gives rise to its wide range of biomechanical responses to load, or how preconditioning perturbs the hierarchical structure of connective tissue. It is only by quantifying responses to load at multiple length scales that we can start to elucidate the structure-function relationships in collagen, the cause of dysfunction in pathology, and the process by which tissue function may be therapeutically restored.

In this study we characterise the tropocollagen spring mechanism by applying well-defined transient strains to strips of human corneal tissue whilst probing the molecular and fibrillar arrangement of collagen using x-ray scattering. By combining these techniques with tissue-scale strain-mapping, a technique utilizing digital image correlation (DIC), we were able to quantify the extent of tropocollagen spring stretch and fibril uncrimping separately. The loading protocol mirrored that of a preconditioning experiment in order to make the results relevant to a wide cross-section of the biomechanics community.

## 2 Materials and methods

### 2.1 Specimen preparation

**Fig. 2:**
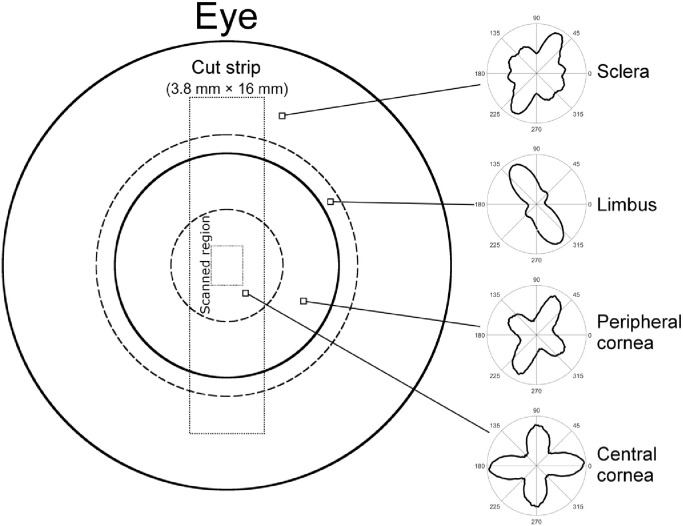
Schematic of the front of the human eye, with the excised strip outlined. The polar plots to the right illustrate the distribution of collagen alignments at the marked points. The limbus and peripheral cornea exhibit circumferential preference about the centre of the cornea, while the central cornea contains collagen biased in the vertical and horizontal directions. The sclera has no overall trend in collagen orientation and is mostly isotropic. Data from [37].

In accordance with the tenets of the Declaration of Helsinki, a total of 14 post-mortem cornea-scleral disks, from human donors aged between 64 and 81 years, were obtained from UK eye banks. The tissue was received following storage in culture medium at a temperature of 37 °C for a period of 2 months. In order to reverse the tissue swelling that occurred during storage and return the corneas to a quasi-physiological hydration and thickness, the organ culture was supplemented with 15% dextran for a period of 2 days before the experiment. Immediately prior to x-ray data collection, the central cornea thickness was measured using an ultrasound pachymeter and subsequently a custom-made cutting device was used to obtain a 3.8 mm × 16 mm strip of tissue from the vertical meridian of each corneoscleral disk (Fig. 2). A diffuse pattern of spray paint was then applied to the strip to create fiducial markers for the strain mapping software to track. As demonstrated previously, spray paint applied in this manner does not affect either the mechanical properties of the tissue or the resulting x-ray images [19,35]. One cornea was used for a preliminary small-angle x-ray scattering (SAXS) study to quantify the extent of the edge effect caused by cutting through collagen fibrils to excise a tensile strip. Of those remaining, seven corneas were assigned to simultaneous SAXS and mechanical testing and six to simultaneous wide-angle x-ray scattering (WAXS) and mechanical testing.

### 2.2 Extensometry

The apparatus has been explained in detail previously [19]. Briefly, cyanoacrylate glue was used to attach the scleral components of each tissue strip to the mechanical clamps of a custom-made extensometer. The extensometer, comprising a piezo motor (Q-545–240, PI, driven by PIShift E-871 controller) and a 4.9 N tension/compression load cell (Model 31, RDP) was then used to apply cyclic strains to the tissue strip, mimicking preconditioning. Each of the 5 cycles involved ramping specimen stress to 500 kPa and then lowering it to 100 kPa, with each cycle taking approximately 1 min (similar to the preconditioning protocol of Boyce et al. [32]). Stress values were calculated based upon thickness measurements at the centre of the cornea and will be slightly less in the thicker periphery and limbus [36]. After the last cycle, the specimen was held at its final length to allow for stress recovery. As the mechanical and structural properties of the cornea are particularly sensitive to changes in tissue hydration, it was necessary throughout the experiment to apply distilled water regularly via an atomizer. Consistency between the specimen weights recorded before and after data collection confirmed this process to be effective in maintaining a close-to-physiological level of hydration.

### 2.3 X-ray scattering

WAXS was carried out on Beamlines I02 and I03 at Diamond Light Source (Didcot, UK), using a series of 0.5 s exposures to an x-ray beam of wavelength 0.979 Å (1.27 keV) and an elliptical beam profile of 50 µm × 90 µm. A Pilatus 6MF detector [38] (Dectris, Switzerland) collected the scattered x-rays at a distance of 0.3 m from the specimen. This setup allowed examination of the molecular arrangement of corneal collagen at length scales ranging from 0.2 nm to 5 nm. The 3.04 Å peak associated with the [104] reflection of calcium carbonate was used to centre and calibrate the images.

SAXS was carried out on Beamline I22 at Diamond Light Source [39], using an x-ray beam of wavelength 1 Å (12.4 keV), with an approximately elliptical profile with major and minor axis lengths 300 µm and 150 µm. The major axis was aligned parallel to the direction of specimen stretch. A Pilatus P3–2 M Silicon pixel detector [38] (Dectris, Switzerland) collected scattered light with an exposure time of 0.5 secs at a distance of 6.0 m from the specimen, with the majority of the scatter path in an evacuated tube to reduce air scattering. This setup allowed examination of the fibrillar architecture of corneal collagen at length scales ranging from 10 nm to 200 nm. The 58.380 Å peak associated with the [001] crystal plane reflection of powdered silver behenate was used to centre and calibrate the images.

To obtain polar distributions of fibrils (using SAXS) and molecules (using WAXS), there are several processing steps that must first be undertaken to identify the centre point of the images and to remove any background signal. This well-established methodology is explained in detail elsewhere [19,40,41]. The regular spacing of collagen fibrils (centre-to-centre distance ∼ 50 nm) gives rise to a broad SAXS peak that is integrated radially to obtain the fibril polar distribution. A similar process is applied to the WAXS peak associated with the tropocollagen intermolecular spacing (approximately 1.6 nm) to obtain the tropocollagen polar distribution. Measurements of d-period (a longitudinal feature of collagen fibrils associated with the crystalline packing of molecules) were made by fitting a cubic spline to the 3rd and 5th meridional SAXS reflections, with the position of the peak value (the statistical mode) being recorded for each. Measurement of the helical pitch of the tropocollagen molecule was also carried out similarly, however as no significant changes were found, these data were omitted.

The extensometer was synchronised with beamline systems to co-ordinate the application of load and the collection of x-ray scatter patterns. During the preconditioning phase, x-ray images were acquired close to the centre point of each specimen at 5 second intervals, meaning an average of 12 images were acquired per cycle. The beamline stage was configured to move the extensometer 150 µm orthogonally to the applied load every half-cycle to avoid repeat exposures, which would increase the magnitude of features associated with photodamage. Following the 5 preconditioning cycles, the strip of tissue was held for a further 10 min to allow it to return close to its equilibrium stress state before acquiring a final scan.

### 2.4 Strain measurement

3D DIC was used to quantify the distribution of strain across the tensile strips as they were loaded. Two CCD cameras (Stingray F-504C, Allied Vision) with ultra-high resolution lenses (50 mm focal length, Edmund Optics) acquired images concurrently with the beamline, so for every x-ray scatter pattern there was a corresponding strain distribution. Istra 4D (Dantec) was used to calculate the engineering strain parallel and perpendicular to the direction of load with sub-pixel resolution. Diffuse fiducial markers provided visual texture for the software to track. A photograph of the apparatus and some example images are shown in the supplementary material.

### 2.5 Statistics

Most of the data described in this study are means of several measurements. We are most interested in the shape of the plots and how far our mean measurements could be from the population mean. As such, statistics shown in the text and figures are mean ± standard error unless stated otherwise.

## 3 Results

### 3.1 Macroscopic biomechanical response

**Fig. 3:**
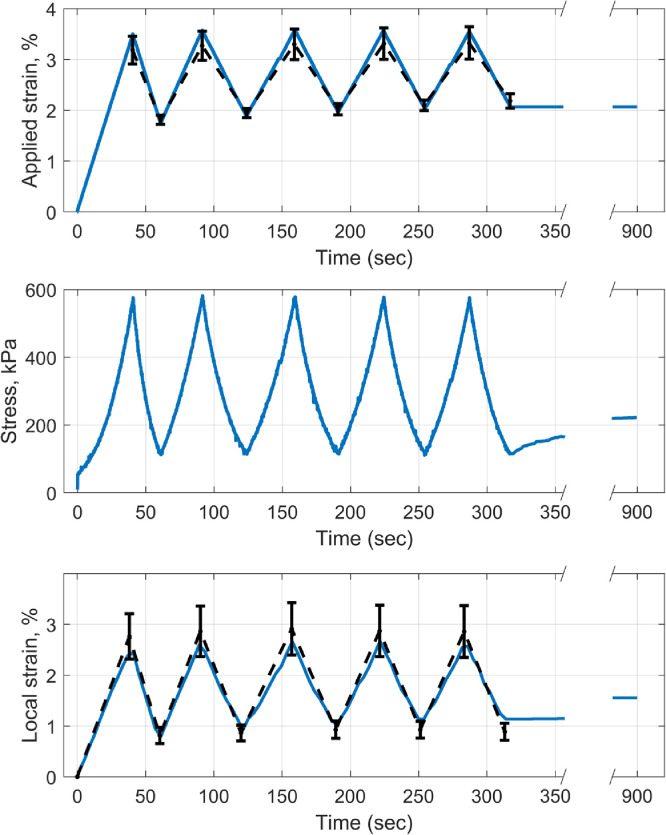
The applied aggregate strain (top) and associated stress (middle) for a representative tissue strip during cyclic loading and subsequent equilibration. The local strain (bottom) was measured within the central 2 mm of the corneal strip, which was where structural data was acquired. Error bars joined by black dashed lines show the population mean and SD. (For interpretation of the references to colour in this figure legend, the reader is referred to the web version of this article.)

Stress and strain data from a single, representative specimen are shown in Fig. 3. The applied strain required to achieve a stress of 500 kPa during the first cycle of loading was 3.2 ± 0.3%, and in the last cycle it was 3.3 ± 0.3% (mean ± SD). With each successive cycle the applied strain required to lift the stress from 100 kPa to 500 kPa reduced, indicating that the cyclic loading stiffened the specimens. Averaged DIC measurements from the central cornea, the region where fibrillar and molecular measurements were acquired, showed the applied stress gave rise to a strain of 2.7 ± 0.9% in the first cycle, rising to 2.9 ± 1.0% in the third cycle before dropping to 2.8 ± 1.0% in the last cycle (mean ± SD). The strain in the centre of the specimen was less than the applied aggregate strain because the central cornea is stiffer in the direction of load than the peripheral cornea and limbus (which have a greater proportion of collagen being aligned radially).

**Fig. 4:**
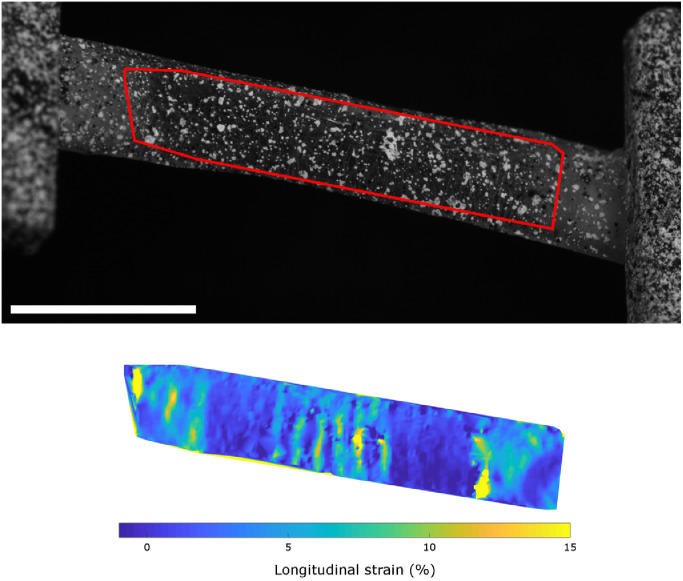
Representative photograph of a stretched tensile strip with fiducial spray paint markers (top) from which the distribution of strain parallel to the direction of applied load was calculated (bottom). Bar 5 mm (approximate due to 3D image).

The local distribution of strain parallel to the direction of load (henceforth referred to as “parallel strain”) was highest in the peripheral cornea, lowest at approximately 2.5 mm from the centre (in a region referred to as the para-centre, and documented as being particularly stiff [36]) and of an intermediate value in the centre (Fig. 4). A striped pattern of parallel strain was seen in the central cornea, which we believe to be associated with the anterior surface crinkling when straightening the tissue strip. This crinkling was not observed when viewed from the posterior side. To account for this surface crinkling effect, local strains were averaged over a 1.5 mm region before being used in calculations of tropocollagen spring stretch and fibril uncrimping (the straightening of fibril waviness).

### 3.2 Quantifying tropocollagen spring stretch and fibril uncrimping

**Fig. 5:**
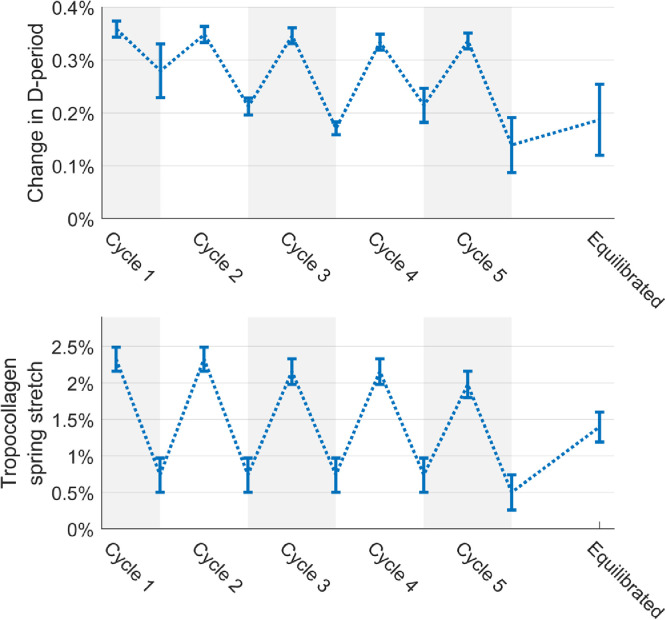
Comparison of the change in d-period over each loading cycle (top) with the change in tropocollagen spring stretch (bottom). Both techniques measure longitudinal fibril strain, but in different ways, and it has recently been suggested that d-band strain underestimates fibril strain [42]. A dotted line has been fitted to the data points to highlight the changes in each parameter with successive cycles.

Fibril stretch can be estimated in two ways – either by measuring changes in d-period (a repeating longitudinal feature of fibrils), or by estimating how much the tropocollagen spring structures have stretched. As a longitudinal fibril feature, changes in d-period infer changes in fibril strain. Fig. 5 (top) shows the average percentage change in d-period from its resting configuration at each peak and trough of the loading cycle. The overall shape of the data is similar to that of the larger-scale measures of strain, although there is also a downward trend in both peak and trough strains that is significant (*p*<0.05, using Pearson's correlation coefficient). The magnitude of the change in d-period is, however, small compared to the local strain and predictions of fibril strain based upon changes in tropocollagen spring stretch although it has been suggested recently that changes in d-period are significantly less than collagen fibril strain in tension [42]. In the direction orthogonal to the applied load, the pattern in the change in d-period is inverted (data not shown).

The process by which tropocollagen spring stretch is measured using x-ray techniques has been discussed previously [19]. Briefly, the polar distributions of fibrils and molecules at a given point in a cornea are different, because tropocollagen molecules are arranged quasi-helically along the axis of their respective fibril. Assuming a helical geometry, the pitch of the helix can be found by convolving the polar distribution of fibrils with the predicted scatter pattern of a helix to obtain a predicted polar distribution of molecules. This can then be compared with the observed polar distribution of molecules, and the modelled twist angle adjusted until the model fits the data. Measurements of supramolecular twist during cyclic loading exhibited a similar trend to that of the d-period (Fig. 5), but several times larger. A key difference between the two measurements was that the twist angle in the troughs remained constant over cycles 1–4 and only showed a small decrease in the 5th cycle, in contrast to the downward trend in d-period. The axial lengthening of a fibril required to confer the maximal change in twist angle (peak 1) was 2.3%, while for the minimal change (trough 5) it was 0.5%.

**Fig. 6:**
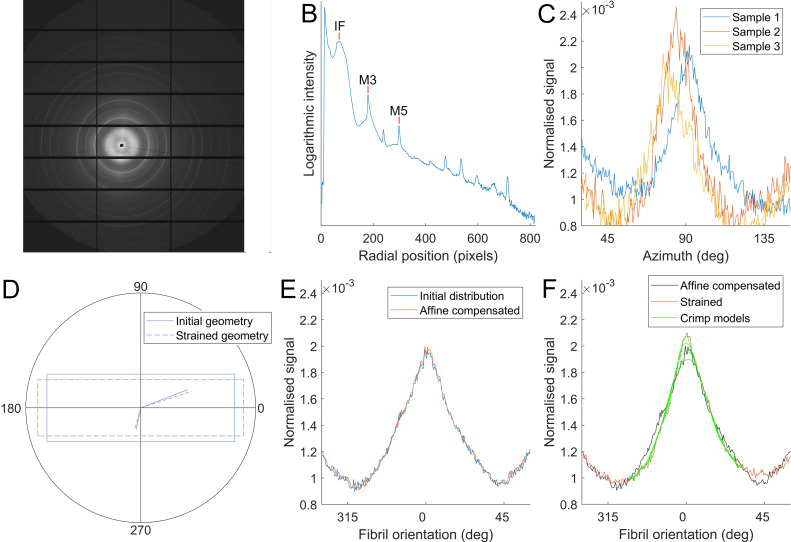
Calculating fibril azimuthal distributions and approximating the effect of changes in gross geometry. A. Example SAXS pattern from the central cornea. B. Radial intensity plot based on A, with interfibrillar (IF) and meridional (M3, M5) peaks labelled, which are used to calculate fibril orientation and d-period, respectively. C. Orientation plots from three representative samples. Orientation plots were rotated about the azimuth until peaks aligned prior to averaging. Samples misaligned by more than 10° were not included in the analysis. D. Averaged initial (unloaded) fibril distribution and a strained distribution. E. Illustration of the principle of affine geometry changes on orientation. Stretch in x and compression in y will cause the orientation of features to change, represented here as solid lines reorientating to dashed lines. F. Using local strain information from DIC, the initial fibril distribution is transformed to approximate the change due to affine changes in tissue shape.

The contribution to the observed total strain associated with the straightening of fibrillar crimp can be estimated by comparing the polar distribution of fibrils before and after a load is applied. The calculation comprises two steps. Firstly, the affine effect of local strains $\varepsilon_{x}$ and $\varepsilon_{y}$ on the initial polar distribution of fibrils, $F_{\text{initial}}\left(\phi \right)$ is calculated to obtain $F_{\text{affine}}\left(\phi \right)$ (Fig. 6). Determination of $F_{\text{initial}}\left(\phi \right)$ was based upon the polar distribution of the interfibrillar SAXS peak (based upon the lateral spacing of fibrils, with a modal average of approximately 50 µm), which was summed over every sample to produce a population average. The averaging process required prior alignment of the peaks; peaks from different samples were not perfectly aligned with the direction of applied load due to natural inter-sample variability and small errors in the alignment of the strip punch. A deviation of less than 10° was deemed satisfactory.

Secondly, assuming negligible fibril slippage, any differences between $F_{\text{affine}}\left(\phi \right)$ and the observed fibril distribution $F_{\text{stretched}}\left(\phi \right)$ (calculated in the same way as $F_{\text{initial}}\left(\phi \right)$ for each set of orientation data in the stretched regime) must therefore be associated with straightening of crimp. Assuming fibrils crimp sinusoidally, the effect of crimp can be estimated by the convolution:

$$F_{\text{stretched}}\left(\phi \right)*F_{\text{sinusoid}}\left(\phi \right)\;∼\;F_{\text{affine}}\left(\phi \right)$$

**Fig. 7:**
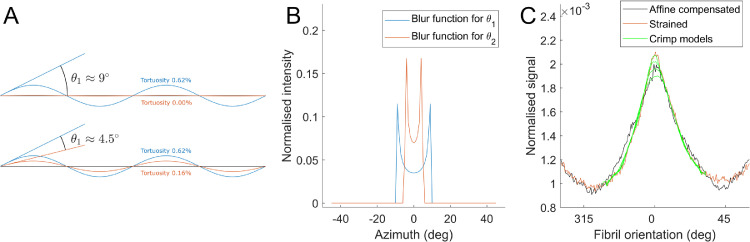
Approximating the extent of collagen fibril uncrimping. A. Illustration of wavy collagen fibrils, showing how a change in crimp angle can lead to a change in tortuosity of the fibril. B. By considering the azimuthal distribution of the sinusoid with a crimp angle equal to the predicted average change of crimp angle in the sample, a blur function can be generated. C. The blur function is convolved with the fibril orientation distribution in the loaded state, and the result compared with the initial fibril orientation distribution that has been corrected for affine changes in sample shape. A best fit is then chosen and a corresponding change in tortuosity calculated. (For interpretation of the references to colour in this figure legend, the reader is referred to the web version of this article.)

Where $F_{\text{sinusoid}}\left(\phi \right)$ is the polar distribution corresponding to an uncrimping fibril (or group of fibrils), which is used as a blur function for $F_{\text{stretched}}\left(\phi \right)$ that can be tuned until a fit with $F_{\text{affine}}\left(\phi \right)$ is achieved (Fig. 7). The blur function widens as the change in crimp angle (the angle of maximum gradient of the sinusoid) increases. This assumes that fibrils uncrimp by reducing their amplitude only, and so for a fibril that traces a path Asin(x) and uncrimps to Bsin(x) (B<A), $F_{\text{sinusoid}}$ would correspond to the azimuthal distribution of the sinusoid (A−B)sin(x). Changes in crimp period were not considered because at low tortuosity (the ratio of straightened length to crimped length, ∼0.5% in the central cornea [13]) the period change in elongation is small in comparison to amplitude change, and by ignoring changes in period the phase is maintained, which reflects the behaviour of a structure embedded in matrix. The tortuosity change due to the change in crimp amplitude then provides an estimate of the contribution fibril uncrimping makes to the local strain. An initial crimp angle of 9° (the angle corresponding to the maximum gradient of the sinusoid), with an associated tortuosity of 0.62% (calculated discretely) was chosen as the initial value. This is slightly higher than literature tortuosity values of 0.5% (corresponding to a crimp angle of ∼8°) which were insufficient to account for the changes in polar distribution between the initial distribution and the strained distribution at the peak of the 5th cycle. We are interested in the uncrimping of fibrils as a longitudinal strain mechanism, and while there will be an opposite “over-crimping” effect in the orthogonal direction as strips contract under load, this is not useful to the study and therefore not measured.

**Fig. 8:**
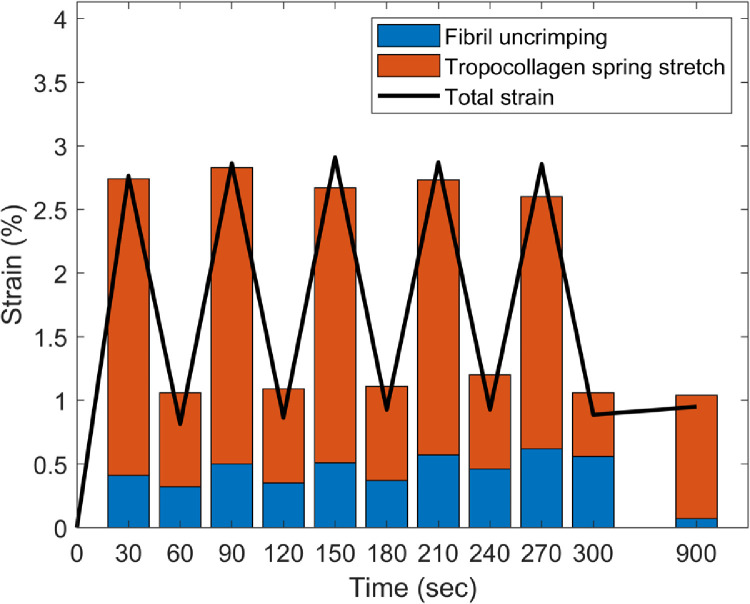
Comparison of local strain parallel to the direction of applied load (line) and the sum contributions of fibril uncrimping (or fibril straightening) and tropocollagen spring stretch (or molecular straightening) during five pre-conditioning cycles and following equilibration.

The sum of the individual contributions of tropocollagen spring stretch and fibril uncrimping are compared with the local tissue strain parallel to the applied load in Fig. 8. The summed contribution resembles the local strain, suggesting that the two mechanisms account for most of the strain in this loading regimen. The overall trend is captured, although there is a gap at the peaks between the total tissue strain and the sum of the two deformation mechanisms that increases with subsequent cycles. There are likely to be several reasons for this discrepancy, not least the geometric assumptions underpinning the models of tropocollagen spring stretch and fibril crimp straightening. Firstly, for reasons of precision, x-ray data was averaged over 5 second periods, and so the data shown in the histograms corresponds to measurements taken during the 2.5 s either side of the corresponding peak or trough. Secondly, there is likely to be an unpredictable amount of fibril slippage, which would have a compound effect over successive cycles. Lastly, given the high power of the x-ray beam used, it is likely that the ∼50 exposures within the ∼3 mm^2^ region caused some very localised tissue drying that would have a progressive effect during the experiment. While changes in gross stiffness due to drying will not affect the analysis, changes in water content and partitioning between the intra- and inter-fibrillar compartments, such as occur during drying, may affect both deformation mechanisms in unpredictable ways. However, based on the known relationship between corneal hydration and collagen intermolecular spacing [43], and the absence of any significant change in the latter during the course of this experiment, it is reasonable to assume that the predicted localised drying caused by the x-ray beam had negligible impact on water content and partitioning. The two mechanisms investigated here behave differently over the course of the loading protocol, with the tropocollagen spring mechanism closely following trends in local strain, while the extent of fibril uncrimping increased with successive cycles, and subsequently decreased during relaxation.

## 4 Discussion

A mechanism by which collagen fibrils can elongate, involving the spring-like stretching of supramolecular structures, has been quantified in a simple stretching experiment. Under cyclic loading of up to 500 kPa, tropocollagen spring stretch accounted for more of the local strain than fibril uncrimping (the straightening of fibril crimp). The results in Fig. 8 contain features that imply the tropocollagen spring mechanism is predominantly elastic, while fibril uncrimping exhibits both elastic and viscous behaviour. These include the near repeatability of tropocollagen spring stretch over successive cycles in contrast to the progressive increase in fibril uncrimping; and following the last cycle, when strain was fixed and stress was allowed to recover, tropocollagen spring stretch increased while fibrils re-crimped. Given that fibril uncrimping requires the lateral movement of a relatively much larger structure through interstices occupied by water and proteoglycans, it would seem logical that this mechanism would involve more energy loss than tropocollagen spring stretch. It is possible that the two processes may drive one another, as the twist direction of the tropocollagen springs is opposite to the direction that unloaded collagen fibrils naturally coil [44]. This means that under tension, the propensity to untwist at one hierarchical level is opposed at the next. The period of stress recovery after the last preconditioning cycle may exemplify this: stress in the tissue strip increased as fibrils recoiled into a more crimped state, thereby applying additional stretch and torque to the molecules (see Figs. 4 and 8).

The use of DIC ensured that aspects of the modelling that incorporated strain measurements reflected the tissue strain in the imaged region only, meaning that corrections for sample thickness or regional stiffness were not necessary. The strain distributions along the tensile strips are in broad agreement with measurements of regional stiffness from the literature, which suggest in meridional tension the central cornea is stiffer than the periphery, which in turn is stiffer than the limbus [36]. However, given that it was not possible to measure changes in sample thickness during the experiment, we cannot provide stiffness measurements of our own. The central cornea, despite being slightly thinner, is known to be significantly stiffer than the periphery and limbus [36], which explains why the local (central corneal) strain was lower than the aggregate strain. The pattern of crinkling across the corneal surface, which was attributed to the straightening of corneal curvature, is a phenomenon that does not seem to have been reported previously, although the biomechanical effect of this straightening has been quantified [45]. The observed straightening of the corneal curvature gives rise to compressive strains on the anterior side and tensile strains on the posterior side. Although it was not possible to separate the effect of these different strains on corneal ultrastructure due to the x-ray scatter technique providing an average, through-thickness measurements of each collagen parameter, the impact of these varying depth-dependant strains was minimised by using the straightened (but un-stretched) corneal tissue strip as a reference point when characterising the average behaviour of each strain mechanism.

**Fig. 9:**
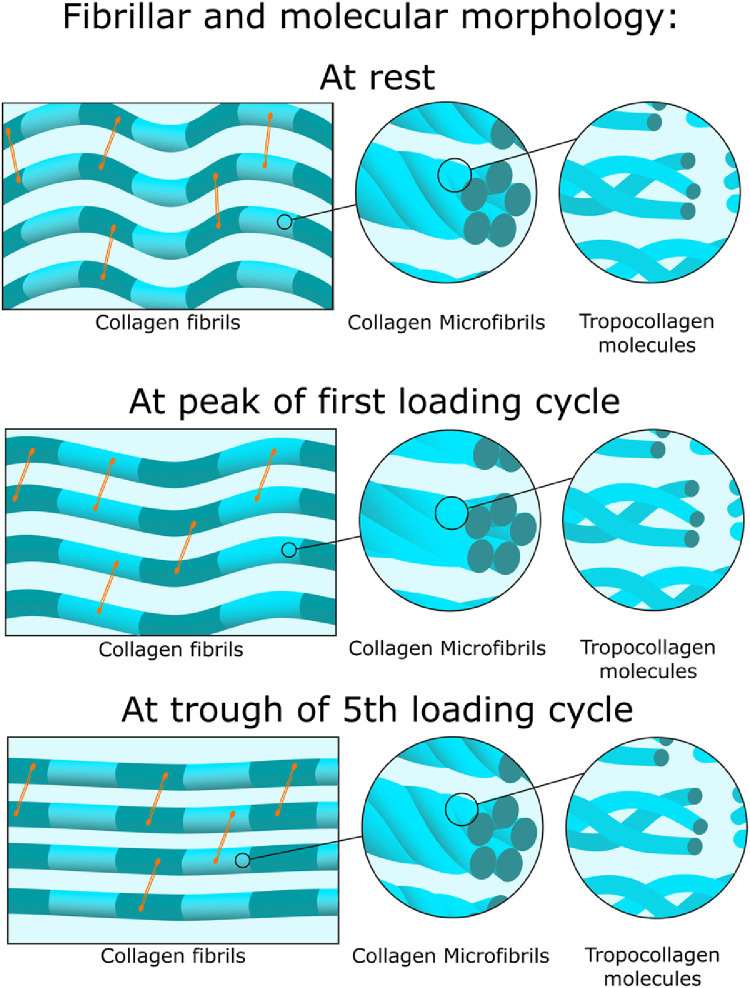
Hierarchical morphology of the collagen network at different stages of the experiment. Prior to the application of load the fibrils are crimped (wavy in appearance) and the d-period (depicted by striped patterns on the fibrils) is a rest value. The microfibrillar structure shows tropocollagen molecules twisted about one-another like springs, with a twist angle of approximately 16° At the peak of the first cycle of load, the fibrils are stretched such that the d-period increases by 0.36 ± 0.02% and the fibrillar crimp is moderately reduced. The fibril stretch is associated with the spring-like straightening of tropocollagen molecules (twist angle reduced to approximately 11°), as can be seen in the microfibrillar architecture, however the tropocollagen triple helices are not affected. At the end of the five loading cycles the fibrils are experiencing a small stretch (D-period 0.14 ± 0.05% higher than rest) and are almost fully uncrimped. This stretch is associated with a slight spring-like straightening of tropocollagen molecules compared to the rest configuration (a twist angle of approximately 15°), while the tropocollagen triple helices remain unaffected (right column). Orange lines represent the proteoglycan network that forms bridges between adjacent collagen fibrils [49]. (For interpretation of the references to colour in this figure legend, the reader is referred to the web version of this article.)

With the two primary deformation mechanisms quantified, a picture can be built illustrating how a piece of collagenous tissue deforms under load (Fig. 9). The more viscous fibrillar uncrimping mechanism is shown in the left column, while the more elastic tropocollagen spring mechanism is shown in the middle column, and manifests as an increase in the d-period in the left column. While the existence of a distinct microfibrillar structure remains a topic of debate it is well-established that tropocollagen molecules are coiled [23,24,46]. A microfibrillar arrangement was chosen in Fig. 9 for simplicity, although the coiled arrangement of tropocollagen molecules is likely to be more complex. It has been suggested that the supramolecular twist angle may increase with radial position within the fibril [27,47] and this is supported by thermodynamic modelling as well as the fact that measurements of surface twist angle acquired by SEM techniques [25,46,48] are generally higher than those taken through the entire fibril, using TEM or x-ray scattering [19,23]. Measurements of molecular conformation in this study showed no changes above the noise level, and for completeness this is shown in the right column.

The tropocollagen spring mechanism may be of specific importance to the cornea, where the quasi-crystalline arrangement of collagen fibrils is vital for maintaining transparency. Strains caused by changes in intraocular pressure will perturb this arrangement less if the fibrils can stretch rather than straighten. The effect of pressure changes on vision are further mitigated by the arrangement of fibrils across the cornea [14], which has the effect of focusing strain away from the refracting central region and into the periphery where it is mediated in part by elastin and fibrillin structures [50]. Given that the eye as a whole experiences pressure fluctuations with timescales ranging from hundredths of a second to hours [8], physiological mechanisms that provide compliance over these timescales would mitigate their effects. An elastic mechanism such as tropocollagen spring stretch is likely to be the predominant response to blinking, eye movements and ocular pulse, while the more viscous uncrimping of fibrils is likely to act predominantly in response to postural changes and circadian rhythm. It would require further study with experiments at these timescales to verify this.

At the end of the experiment the specimens were near equilibrium and the extent of fibrillar uncrimping relative to the unloaded state was very small. The re-crimping of fibrils during the equilibration phase resulted in the stress increasing (see Fig. 3), which is viscous behaviour and implies the existence of internal forces that drive re-crimping. This may explain why Liu et al. reported needing static strains of up to 20% to fully straighten corneal collagen fibrils [51] despite the measured tortuosity being several times smaller. While the tropocollagen spring mechanism cannot account for the huge strains in Liu's study (such large strains require stresses sufficient to cause significant fibril slippage and partial rupture), these findings would suggest that fibril crimp contributes more to a tissue's dynamic mechanical properties than its static mechanical properties, as would be expected of a viscous mechanism. The precise extent of this contribution will require a careful multimodal approach to elucidate, it is known, for instance, that age influences stiffness and crimp metrics [52]. In this study we used specimens with as small an age range as possible (64–81) to minimise this variability.

The results of this study also provide some insight into the hierarchical changes caused by preconditioning tissue prior to a mechanical test. Given that immediately after a series of loading cycles the crimp has been mostly removed, any subsequently applied load will be resisted largely by the tropocollagen spring mechanism and other higher strain mechanisms such as slippage, depending upon the magnitude. While there are undoubtedly other changes that occur during preconditioning, such as reorientation of collagen not associated with uncrimping, the removal of fibril crimp will make tests less viscous and thus easier to control and model.

Although every effort was made to minimise sources of error in the study, there were some unavoidable limitations. Firstly, neither tropocollagen spring mechanism nor fibril crimp will be perfectly described using trigonometric functions, and while the spring mechanism is measured in 3D, we assumed a fibril waviness to be 2D so as to be compatible with literature measurements of tortuosity. Secondly, assumptions that sinusoidal fibrils uncrimp through changes in amplitude only will under-estimate the extent of fibril reorientation under load (leading to an over-estimate of the extent of fibril uncrimping), although the magnitude of this error is likely to be small.

## 5 Conclusion

In conclusion, we have shown that the tropocollagen spring mechanism endows collagen fibrils a means of elongating elastically under small strains. Through a modelling approach we also quantified the extent to which fibrils simultaneously uncrimp, and found the uncrimping mechanism to be significantly more viscous. The ability of some collagen fibrils to elongate reversibly has significant implications for the understanding of connective tissue biomechanics, and the effects of pathology and associated treatments. The ability of collagen fibrils to stretch and recoil whilst crimped means that “hook on” models (common in arterial research, which imply collagen fibrils only confer resistance to load when fully straightened, see [53]) may not be accurate. Connective tissue pathologies often lead to the laying down of thicker fibrils (e.g. diabetes [54], fibrotic intervertebral disc degeneration [55]) or thinner fibrils (e.g. hypertension [56], wound healing [57]), which are likely to have different twist angles to native healthy tissue and therefore different abilities to elongate via the tropocollagen spring mechanism. Tissues with an impaired tropocollagen spring mechanism would be more reliant on fibril uncrimping in order to distend, making their response to load more viscous. A better understanding of the deformation mechanisms that occur in health and disease may help in the design of targeted treatments aimed at treating connective tissue dysfunctions. For instance, methods of artificially stiffening weakened connective tissue (such as the use of photochemical crosslinking to stiffen surgically or pathologically weakened corneas [58]) may derive their effect from a restriction of one or both of the mechanisms measured in this study. The co-existence of tropocollagen springs and fibrillar crimp in many tissues throughout the mammalian body suggests they are likely to have important physiological roles. There already exist comprehensive biomechanical models of fibril crimp, such as [11], which currently assume fibril inextensibility. It would be of tremendous value to incorporate fibril elongation via the tropocollagen spring mechanism to these models, as an approach to identify its physiological roles. We are only beginning to understand the precise nature of these structures, and further research is required to elucidate their function in health, their dysfunction in pathology and methods for therapeutic intervention.

## Declaration of Competing Interest

The authors declare that they have no known competing financial interests or personal relationships that could have appeared to influence the work reported in this paper.
